# Drivers of cocoa agroforestry adoption by smallholder farmers around the Taï National Park in southwestern Côte d’Ivoire

**DOI:** 10.1038/s41598-023-41593-5

**Published:** 2023-08-31

**Authors:** Jean-Luc Kouassi, Lucien Diby, Dieudonné Konan, Allegra Kouassi, Yeboi Bene, Christophe Kouamé

**Affiliations:** 1 DFR Eaux, Forêts et Environnement, Institut National Polytechnique Félix Houphouët-Boigny (INP-HB), BP 1093, Yamoussoukro, Yamoussoukro Côte d’Ivoire; 2 International Cocoa Organization, ICCO Building, II Plateaux ENA-Avenue Boga Doudou, 06 BP 1166, Abidjan 06, Abidjan Côte d’Ivoire; 3 World Agroforestry (CIFOR-ICRAF), Côte d’Ivoire Country Programme, 08 BP 2823, Abidjan 08, Abidjan Côte d’Ivoire; 4 Projet de Développement des Chaines de Valeur Vivrières (PDC2V), Ministère d’Etat, Ministère de l’Agriculture et du Développement Rural, 06 BP 496, Abidjan 06, Abidjan Côte d’Ivoire

**Keywords:** Sustainability, Climate-change ecology

## Abstract

The encroachment of agricultural expansion into protected areas has led to severe biodiversity loss. To promote sustainable agriculture practices and reverse the anthropogenic pressure, several initiatives such as the Cocoa and Forests Initiative (CFI) and the National Strategy for Reducing Greenhouse Gas Emissions from Deforestation and Forest Degradation (REDD+), have been undertaken. This study examines the adoption of cocoa agroforestry by smallholder farmers in the vicinity of the Taï National Park (TNP) in Southwestern Côte d’Ivoire. A structured questionnaire was administered to 323 cocoa farmers to understand their practices and perceptions of cocoa agroforestry. Results showed that most farmers (95%) grow unimproved cocoa varieties with an average yield of 376 ± 36 kg ha^−1^ year^−1^. The majority of farmers (86%) use agroforestry practices in their farming systems, with pruning techniques being used by 82% and fertilizers applied by 27%. Additionally, 54% of farmers are adopting improved agroforestry practices or planting more trees in their cocoa plantations. Factors influencing cocoa agroforestry adoption include gender, the length of residency, the number of cultivated cash crops and the incidence of black pod attacks. These findings highlight the potential to leverage community knowledge in promoting sustainable agricultural practices and generate positive impacts. These results have important implications for future initiatives aiming to promote sustainable agriculture practices and biodiversity conservation in the region. By capitalizing on the adoption of agroforestry and leveraging socioeconomic factors, it is possible to enhance the conservation of the TNP and promote sustainable cocoa farming practices.

## Introduction

Côte d’Ivoire is the world’s leading cocoa producer, accounting for approximately 43% of global cocoa production^1^. Cocoa is a vital component of the Ivorian economy, contributing approximately 15% of the country’s Gross Domestic Product (GDP) and generating 39% of the country’s export income^2^. About two-thirds of the active population, including 1 million smallholder farmers, depend on cocoa for their livelihoods^2,3^. Despite this importance, cocoa is still cultivated in extensive traditional cropping systems mainly in primary forests including protected areas^4^. It is estimated that 30–40% of the cocoa produced in the country is derived from protected lands^5^. As a result, cocoa production contributes to over 38% of agricultural-led deforestation in Côte d’Ivoire^6^. Between 1986 and 2020, the cocoa production areas increased from 1.2 to 3.3 million hectares^3,7^, while forest cover dropped by 70% during the same period^8,9^.

The massive expansion of cocoa farms into gazetted forests (GFs) and protected areas has primarily been driven by the search for endogenous fertile soils and the humid microclimate in these forests, which are conducive to improved cocoa growth and production^4,10–12^. Other reasons for the pressure on the GFs include the scarcity of forest lands in the rural areas, the mixed success of cocoa replantation programs, the massive attraction of migrants from both within Côte d’Ivoire and neighboring countries by the cocoa economy and the disruption of GF monitoring during the political crisis^4,13^. The southwest part of the country, home to the Taï National Park (TNP)—a world heritage site and one of the largest undisturbed rainforests and biodiversity gems in West Africa—is particularly threatened by these challenges.

Migration around the TNP has occurred in multiple waves. The first wave in the 1970s took place in the regions of Buyo in the northeast of the TNP and involved Ivorian migrants, mainly Baoulé people, who were displaced by the construction of the Kossou Dam^14^. The second wave in the 1980s consisted mainly of non-Ivorian migrants mainly from Burkina Faso, settling in the west and south of the TNP and its surrounding GFs^4,15^. The third wave occurred during the political and military crisis from 2002 to 2011, resulting in an unregulated influx of foreign migrants into the protected zones^4,14^. These migrations have intensified pressure on forest lands and exacerbated land-related issues.

The high level of cocoa production and its impact on deforestation have led to several initiatives to mitigate encroachment into GFs and protected areas. For example, the cocoa industry under the leadership of the governments of Côte d’Ivoire and Ghana have launched the Cocoa and Forests Initiative (CFI) in 2017 to alleviate cocoa-driven deforestation and promote cocoa agroforestry. In 2018, the country adopted the National Strategy for Reducing Greenhouse Gas Emissions from Deforestation and Forest Degradation (REDD+) and the Forest Preservation, Rehabilitation and Expansion Policy coupled with its implementing Strategy, which aims at reaching 20% of the country's surface covered by forest by 2030. Additionally, the Government issued in 2022 a Policy Statement and a National Strategy for sustainable cocoa farming, which integrates the fight against deforestation and child labor in the cocoa value chain, as well as the improvement of farmers' incomes. The new forest law and the decree regulating the implementation of the African Regional Standard (ARS) 1000 for sustainable cocoa adopted respectively in 2019 and 2022, have emphasized the implementation of agroforestry in highly encroached GFs and the promotion of cocoa agroforestry in existing farms located in the rural areas. Furthermore, a greenhouse gas emission reduction project is now underway surrounding the TNP, intending to generate 10 million tonnes of reduced emissions by promoting agroforestry for fighting against climate change, diversifying incomes and protecting natural resources.

While full-sun cocoa farming remains widespread, farmers in the southwestern and eastern cocoa belts are adopting of cocoa agroforestry practices. This shift is driven by factors such as erratic rainfall patterns and the promotion of good agricultural practices (GAP) by extension services. This practice involves interplanting various tree species in cocoa plantations, providing benefits such as increased yields and improved soil fertility^16^. Certain shade trees, like *Albizia zygia*, *Milicia excelsa*, and *Glyricidia* spp., have been shown to enhance cocoa productivity^16,17^. Agroforestry systems also contribute to natural pest control^18^, reducing diseases like cocoa swollen shoot virus disease (CSSVD)^19^. Moreover, the diversification of income streams through additional products from interplanted trees, such as fruits, nuts, or timber, contributes to farmers’ livelihoods^20,21^.

Despite the encouraging trend highlighted above, more effective sensitization strategies must be developed to enhance the presence of trees in cocoa farms on a broader scale. Identifying the factors that affect cocoa agroforestry adoption is crucial for promoting its widespread implementation. On-farm practices and issues, such as the application of GAP including pruning, as well as the presence of pests and diseases such as CSSVD, play a significant role in facilitating agroforestry adoption by optimizing shade levels and creating a favorable environment for integrating trees in cocoa farms^18,22,23^. In addition to on-farm practices, several other factors influence cocoa agroforestry adoption. Gender dynamics play a role, as women may have different levels of access to resources and decision-making power, which can affect their engagement in agroforestry practices^24^. Age and position within the household may also influence the willingness and ability to adopt agroforestry systems^25^. Moreover, experience in agriculture, the duration of residency and extension contact can impact farmers’ perception of risk and their willingness to adopt agroforestry as a resilience strategy^26–29^.

Past studies investigating cocoa agroforestry adoption in the context of cocoa production in Côte d'Ivoire have undoubtedly shed light on some critical factors affecting of adoption of sustainable cropping systems^11,25,30^. However, it is worth noting that many of these studies have focused primarily on socioeconomic indicators and access to agricultural inputs including tree seedlings^11,25,30^, overlooking the significance of on-farm agricultural practices and the proximity of cocoa plantations to the TNP in shaping farmers’ decisions to adopt agroforestry systems. Despite the potential significance of agricultural practices in agroforestry adoption, they have not received adequate attention in previous research efforts. Furthermore, the proximity of cocoa plantations to the TNP and other protected areas introduces a unique dimension to the cocoa agroforestry adoption equation. The favorable weather conditions and endogenous fertile soils found in these forests are well-known for their positive impact on cocoa growth and production. As such, farmers with plantations in close proximity to the TNP may have different motivations and incentives to adopt agroforestry systems compared to those situated farther away. The existence of migratory patterns around the TNP^4,14,15^, with multiple waves of migration driven by various factors, can also contribute to changes in land use and intensify pressure on forest lands. Therefore, in order to gain a more comprehensive understanding of cocoa agroforestry adoption and its potential contribution to TNP conservation, it is essential to explore the interplay between socioeconomic indicators, on-farm agricultural practices, and the geographical proximity of cocoa plantations to the TNP. This study aims to address these gaps in the literature and provide a more nuanced perspective on the drivers of cocoa agroforestry adoption in the peripheral zone of the TNP in the southwestern region of Côte d’Ivoire. To achieve this, socioeconomic considerations affecting agroforestry adoption were investigated, agricultural practices that lead to agroforestry adoption were mapped out, and factors that influence farmers' opinions about adopting agroforestry were determined. The hypotheses tested are threefold: (1) socioeconomic indicators influence agroforestry adoption; (2) farmers’ perceptions and adoption of agroforestry vary in relation to the position of their plantations to the TNP; and, (3) low cocoa plant density and low incidence of pests and disease infection drive farmers to adopt agroforestry. By considering these multifaceted factors, we can better inform policy and conservation efforts and contribute to sustainable cocoa production and forest preservation in the region.

## Material and methods

### Study area

**Figure 1:**
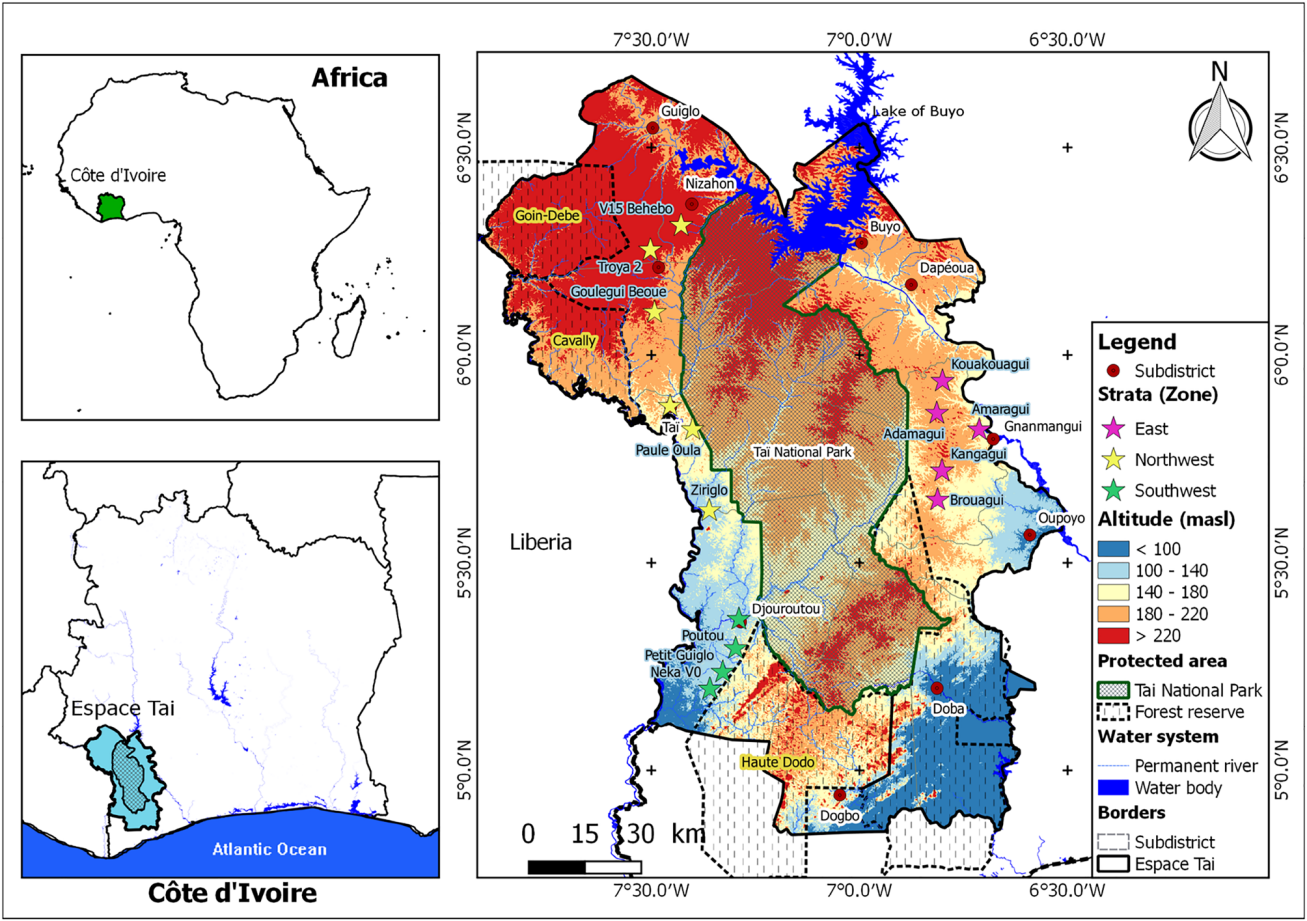
Map of the study area showing surveyed localities.

The study was conducted in the “*Espace Taï”,* the wider area surrounding the TNP, the largest protected rainforest in West Africa and a UNESCO World Heritage site since 1982 (Fig. 1). This area spans over 17,334 km^2^ and is located in southwestern Côte d'Ivoire between latitudes 4°50′ and 6°42′ North and longitudes 6°30′ and 7°56′ West.

The *Espace Taï* comprises 11 sub-districts across Nawa, Cavally, and San-Pedro regions and has an estimated population of 842,640 inhabitants, with about half of the population consisting of migrants from neighboring countries^31^. The average rainfall is 1700 mm but varies from 1500 mm in the northeast to 2200 mm in the southwest^32^. The TNP is surrounded by five GFs, which are mostly degraded by cocoa cultivation from migrant farmers, except for the GF of Cavally in the northwest, which is less infiltrated and occupied by cocoa^33–35^. With a high population of migrants (national and neighboring countries), the east of TNP is generally more densely populated than the northwest and southwest regions^31^. The region to the east is closer to major cities such as Buyo and Meagui, and is also more accessible by road. As a result, this region has experienced more rapid population growth, urbanization and cocoa production in recent years. On the other hand, the areas to the northwest and southwest of the park are more remote and have historically had lower population densities and highly composed of autochtonous communities^14,31^. These regions are largely rural and dominated by perennial and subsistence agriculture, with many communities relying on the park’s resources for their livelihoods. In terms of land use, the eastern landscape is mainly composed of cocoa plantations, while the northwest is occupied by rubber and cocoa and the southwest by palm oil and cocoa^34^.

### Household survey

#### Sampling techniques

To ensure a representative sample for the household survey, localities and households household were selected using a stratified sampling technique based on the geographical characteristics of the study area. The three geographical strata*,* namely East, Northwest, and Southwest, were determined taking into consideration the population composition and documented agricultural practices. Within each stratum, 4–6 localities, with more than 1200 inhabitants, were randomly selected for the survey (Fig. 1). In total, 323 households were randomly selected for interviews. The sample size was calculated by Fisher's formula at the 95% confidence interval and a ± 5% margin of error^36,37^.

1 $$n = \left[ {\frac{{Z^{2} }}{{E^{2} }}p\left( {1 - p} \right)} \right]$$

where:

- *n* is the required sample size.
- *Z* is the z-score corresponding to the desired confidence level (Z = 1.96 for 95% confidence interval);
- *p* is the estimated proportion of the population with the characteristic of interest;
- *E* is the desired margin of error (0.05).

To ensure that households selected were representative of the population in each stratum, a probability-proportional-to-size (PPS) sampling technique was employed. The number of households selected from each locality was proportional to the population size of the locality. Within each locality, 11–30 households were randomly selected to achieve a suitable sample size. The survey questions were designed to capture a wide range of agricultural practices and adoption opinions, and the data collection process was conducted rigorously using the Census and Survey Processing System (CSPro) mobile data collection application. The household survey was conducted with trained enumerators who received comprehensive training on ethical considerations and the appropriate use of survey tools. The average duration of the survey was estimated at 30 min.

#### Ethical considerations

Before starting the survey, informed consent was obtained from all participants, and participants were informed that their responses would be kept confidential and only used for research purposes. In addition, the survey did not collect any identifying information from respondents.

The experimental protocol and information to be collected were reviewed and approved by the Autorité de Régulation des Télécommunications de Côte d'Ivoire (ARTCI) under the license number 2017–0279, which served as institutional ethics and licensing committee for surveys.

Any sensitive questions, such as those related to income or land ownership, were asked at the end of the survey to minimize discomfort for respondents. Finally, enumerators were trained to handle any distressing situations or ethical concerns that may arise during the survey.

These measures were taken to ensure the ethical conduct of the study in accordance with relevant guidelines and regulations and to protect the welfare and privacy of the participants.

#### Data analysis

Mean, frequency, and percentage provide insight into agricultural practices and adoption opinions. Given the non-normal distribution, significant differences between responses of variables and their relationship to the TNP were assessed using the Kruskal–Wallis test for quantitative variables. The Bonferroni adjustment was used as a differentiating post-hoc test. On the other hand, a Pearson chi-squared test at a 95% confidence interval was used for qualitative variables.

The influence of independent variables on farmers' adoption opinions was determined through binomial logistic regression in the logit model and found variables to explain adoption opinions. We also assessed multicollinearity using diagnostic tests, and only included variables that had a low correlation with each other. Specifically, we used a variance inflation factor (VIF) threshold of less than 2.5 to detect multicollinearity^38,39^ (Table S1). Out of the 24 variables initially considered, including land tenure, wealth, social standing, access to labor, on-farm practices, and other factors, only ten variables exhibited a VIF below 2.5. These ten independent variables, which met the criteria, were included in the final model. They encompassed gender, age, position of the respondent in the household, duration of residency, number of cash crops, presence of mirids, black pod disease and CSSVD in cocoa farms (Table S1). The model was generated using a backward elimination procedure based on minimizing the Akaike information criterion (AIC). This binary logistic regression showed the probability of the effects of independent variables on dependent variables^40^:

2 $$p{ } = E\left( {Y = 1{| }X_{{i_{1 \le i \le n} { }}} } \right) = { }\frac{1}{{1 + { }e^{{ - {\text{Z}}}} }} = \frac{{{\text{e}}^{Z} }}{{1 + { }e^{{\text{Z}}} }}$$

Since *p* gives the probability of adopting, the probability of not adopting, is:

3 $$1 - p{ } = E\left( {Y = 1{| }X_{{i_{1 \le i \le n} { }}} } \right) = { }\frac{1}{{1 + { }e^{{\text{Z}}} }}$$

Equation (3) expresses the logarithm of the odd ratio in favour of adopting agroforestry practices. This ratio represents the log-transformation of the ratio of the probability that a household will adopt to the probability that it will not adopt agroforestry practices.

Finally, the logit model that is considered is expressed as follow:

4 $$L = \ln \left( {\frac{p}{1 - p}} \right) = a_{0} + a_{1} X_{1} + a_{2} X_{2} + \cdots + a_{n} X_{n} + \varepsilon$$

In all the above equations:

- $p$ is the probability of adopting agroforestry practices.
- *Y* is the dependent binary variable defined as follow: *Y* = 1, if the event occurred (adopted), and *Y* = 0, if the event did not occur (non-adopted);
- ${a}_{0}$ is the constant term (intercept);
- ${a}_{1}$ … ${a}_{n}$ are coefficients of associated independent variables;
- ${X}_{1}$… ${X}_{n}$ are independent variables;
- $\varepsilon$ is the residual term of the logistic regression.

The Average Marginal Effects (AME) were computed to assess the impact of a predictor on the outcome variable^41^. We also used the pseudo R-squared value (Nagelkerke) to estimate the proportion of variance explained by the model. The likelihood ratio test model summary was used to report metrics for the binary logistic regression model fit^40^. Only significant (p-value < 0.05) independent variables were considered having an association with agroforestry adoption. All analyses were carried out using the R statistical software packages *questionr*^42^, *margins*^41^, and *stats*^43^.

## Results

### Sociodemographic and socio-economic characteristics of the farmers

The study sampled 323 cocoa farmers, with 38.4% located in the East, 33.4% in the Northwest, and 28.2% in the Southwest (Table 1). Of the farmers, over 84% were men, between the ages of 18 and 81 years old, with an average age of 40.1 years. The average household was headed by a 45-year-old man and included 8.8 people, with an equal proportion of men and women. Most interviewees were married and had never attended school. Half of the households were migrants from Burkina Faso, Niger, Mali, and Benin, and settled in the region about two decades ago, showing that non-native populations settled before the 2002–2010 sociopolitical crisis of Côte d’Ivoire. The study found no significant difference (p > 0.05) between surveyed zones, except for the gender and original status of respondents (Table 1).

**Table 1:** Sociodemographic characteristics of households (N = 323) in the “*Espace Taï*”.

Sociodemographic attributes		East	North-west	South-west	*Espace Taï*	*p*-value
Household	Proportion (%)	38.4	33.4	28.2	100.0	*n.s. β*
	Duration of residency (year)	25.6	23.8	22.1	24.0	*n.s. β*
	Men (no.)	4.5	4.1	4.9	4.5	*n.s. β*
	Women (no.)	4.4	3.6	4.9	4.3	*n.s. β*
	Size (no.)	9.0	7.4	9.8	8.8	*n.s. β*
	Household head age (years old)	42.9	45.9	45.8	44.7	*n.s. β*
Respondent gender (% of farmers)	Female	10.5	6.5	34.1	15.8	*p* < 0.001 α
	Male	89.5	93.5	65.9	84.2	
Respondent age (% of farmers)	18–30 years old	28.2	34.3	34.1	31.9	*n.s.* α
	30–60 years old	63.7	54.6	61.5	60.1	
	60–90 years old	8.1	11.1	4.4	8.0	
Original status (% of farmers)	Non-native	60.5	18.5	33.0	38.7	*p* < 0.001 α
	Native	3.2	11.1	5.5	6.5	
	Foreigner	36.3	70.4	61.5	54.8	
Marital status (% of farmers)	Single	4.0	7.4	2.2	4.6	*n.s.* α
	Married	93.5	90.7	92.3	92.3	
	Divorced	0.8	0.0	0.0	0.3	
	Widowed	1.6	1.9	5.5	2.8	
Education level (% of farmers)	None	66.9	72.0	81.3	72.7	*n.s.* α
	Primary school	20.2	22.4	12.1	18.6	
	Secondary school	12.1	5.6	5.5	8.1	
	University	0.8	0.0	1.1	0.6	

n.s., non-significant; α, Pearson chi-squared test; *β*, Kruskal–Wallis chi-squared test.

The farmers grew an average of seven different crops, including food and cash crops (Table 2). The size of the cocoa farm ranged from 0.5 to 71 ha, with an average of just over 5 ha. Farmers produced an average of 1.8 metric tons of cocoa beans, representing 376.3 kg ha^−1^ year^−1^ during the years 2013, 2014, and 2015. Within zones, the lowest yield was highlighted in the Northwest while the highest was identified the East. More than half of households had incomes exceeding XOF 500,000 per year, and two-thirds of households did not have other sources of income than agricultural activities.

**Table 2:** Socioeconomic characteristics of cocoa households (N = 323) in the “Espace Taï”.

Socioeconomic attributes		East	North-west	South-west	*Espace Taï*	p-value
Principal source of income (% of farmers)	Farming	99.2	100	98.9	99.4	*n.s.* α
	Trading	0.8	0.00	1.1	0.6	
Number of cultivated crops	Total	7.1^b^	7.5^ab^	8.3^a^	7.5	*p* < 0.05 *β*
	Food crops	5.4	5.2	5.9	5.5	*n.s. β*
	Cash crops	1.7^b^	1.7^b^	2.4^a^	2.1	*p* < 0.05 *β*
Cash crops (frequency in % of farmers)	Cocoa	100	100	100	100	–
	Rubber	25.8	63.6	57.1	47.2	
	Coffee	33.9	58.9	34.1	42.3	
	Oil palm	3.2	5.6	46.1	16.1	
Food crops (frequency in % of farmers)	Rice	78.3	88.8	82.4	82.9	–
	Maize	61.3	80.4	90.1	75.8	
	Cassava	52.4	48.6	70.3	56.2	
	Yam	79.1	37.4	44.0	55.3	
	Eggplant	29.0	38.3	45.0	36.7	
	Okra	30.7	34.6	39.6	34.5	
	Pepper	29.0	31.8	36.3	32.0	
	Plantain	34.7	18.7	33.0	29.0	
Farm size (ha)	Cocoa	5.8	4.4	5.3	5.2	*n.s. β*
Cocoa production (Mg)	2013	2.2^a^	1.6^b^	1.7^ab^	1.9	*p* < 0.05 *β*
	2014	2.45^a^	1.5^b^	1.7^ab^	1.9	*p* < 0.05 *β*
	2015	0.85^b^	0.8^b^	3.9^a^	1.7	*p* < 0.05 *β*
Cocoa yield (kg ha^–1^ year^–1^)	2013–2015	372.2^ab^	297.3^b^	475.8^a^	376.3	*p* < 0.05 *β*
Agricultural activity income (% of farmers)	None	0.0	0.9	0.0	0.3	*p* < 0.05 α
	< XOF^θ^ 500,000	25.0	30.8	36.3	30.1	
	XOF 500,000–1,000,000	32.3	31.8	34.1	32.6	
	> XOF 1,000,000	41.9	26.2	29.7	33.2	
	Do not know	0.8	10.3	0.0	3.7	
Non-agricultural activity income (% of farmers)	None	77.4	86.9	65.9	77.0	*p* < 0.05 α
	< XOF 500,000	17.7	8.4	27.5	17.4	
	XOF 500,000–1,000,000	0.8	0.0	1.1	0.6	
	> XOF 1,000,000	3.2	0.0	0.0	1.2	
	Do not know	0.8	5.6	5.5	3.7	

n.s., non-significant; α, Pearson chi-squared test; *β*, Kruskal–Wallis chi-squared test.

Means within a column with the same letter are not significantly different from each other at the 5% level.

^θ^The West African CFA Franc is assumed to have a fix parity with the Euro at 1 Euro = 655.957 XOF.

### Characteristics of cocoa farms and field practices

#### Cocoa tree density, harvesting period, and frequency

Most cocoa plantations were established at irregular densities through direct seeding of unselected planting materials (Table S2). Only about 13% used improved planting materials from the National Agricultural Services Agency (ANADER) and the National Agricultural Research Center (CNRA). On average, farmers conducted 7.8 manual harvests per year. Approximately 51.2% of households based their harvest schedule on a monthly basis, while 24.5% determined the timing of harvests based on the ripeness intensity. The main harvesting period was slated from September to December (Table S3), with 75% of respondents identifying peak harvest in October.

#### Cocoa farm maintenance

Cocoa orchard maintenance practices including pruning, weeding, fertilization, and phytosanitary and sanitary practices were performed by farmers in the Wider Tai area. Results showed that almost 88% of farmers used suckering, 83% pruned, and about 59% used cutting practices. Very few practiced sanitary harvesting or staking (Table S4). Almost all farmers manually weeded twice a year, while half used chemical herbicides additionally one time per year (Table S5). Over 71% of farmers applied fertilizers on their cocoa farms (Table S6) while 91% of farmers applied pesticides (Table S7). Mirids were observed by over 90% and black pods by almost 88% of farmers (Table S8).

#### Cocoa agroforestry practices

In the region, most of the surveyed farmers (86.2%) grew cocoa under companion trees, with over 75% having fruit trees, and 43.5% having one additional tree species in their cocoa plantations (Table 3). Against about 13.8% of farmers declared to grow cocoa without any companion trees. The cocoa yield was estimated at 353.6 kg ha^−1^ year^−1^ and 398.8 kg ha^−1^ year^−1^ for agroforestry non-adopter and adopter farmers, respectively. The most important functions of companion trees included food provision (food trees and fruit trees) and shade production according to 52% and 36% of farmers, respectively. Fruit trees are characterized by producing fruits that are typically consumed directly (e.g. *Citrus* sp., *Psidium guajava*, *Mangifera indica*, etc.), while food trees yield fruits that are commonly cooked or transformed for culinary preparations (e.g. *Ricinodendron heudelotii*, *Irvingia gabonensis*, etc.). Farmers estimated that the majority of their cocoa agroforestry systems had low (47.4%) to medium (36%) tree density. Regarding tree density and assigned role to agroforestry practices, no significant differences were identified between surveyed zones.

**Table 3:** Cocoa agroforestry practices of households (N = 323) in the “Espace Taï”.

Agroforestry practices		East	North-west	South-west	*Espace Taï*	p-value
Presence of companion tree (% of farmers)	No	7.6	20.6	13.2	13.8	*p* < 0.05 α
	Yes	92.5	79.4	86.8	86.2	
Companion tree type (frequency in % of farmers)	Fruit trees	98.0	68.6	55.7	75.7	–
	Tree species	35.7	37.2	60.8	43.7	
Companion tree density (% of farmers)	Null (full-sun)	2.9	6.2	2.1	3.5	*n.s.* α
	Low (< 10 trees ha^–1^)	48.6	53.1	41.7	47.0	
	Medium (10–20 trees ha^–1^)	34.2	21.9	45.8	35.6	
	High (> 20 trees ha^–1^)	14.3	18.8	10.4	13.9	
Main role of companion trees (% of farmers)	Food	65.7	46.9	45.8	52.2	*n.s.* α
	Shade	20.0	37.5	47.9	36.5	
	Delimitation	2.9	6.2	0.0	2.6	
	Lumber	2.9	0.0	2.2	1.8	
	Fertilization	0.0	3.1	2.1	1.7	
	Firewood	0.0	0.0	2.1	0.9	
	Other	8.6	6.2	0.0	4.3	

n.s., non-significant; α, Pearson chi-squared test.

ANADER provided more than half of farmers with tree planting advice (Table S9). The majority of the farmers (57%) had removed tree species in their plantations due to too much shade and incompatibility or competition between tree species and cocoa trees (Table S10). However, about 54% were positive about adopting agroforestry, whereas 46% rejected agroforestry (Table 4) due to the shortage of arable lands and ignorance of ecosystem services of cocoa companion trees. The desired tree species were mainly food (69%), legumes (66%) and shade (37%) tree species.

**Table 4:** Factors explaining the attitude of farmers towards cocoa agroforestry in the Wider Taï area.

Opinions on adoption of companion trees		East	North-west	South-west	*Espace Taï*	p-value
Companion tree adoption (% of farmers)	No	27.4	46.3	68.1	45.2	*p* < 0.05 α
	Yes	72.6	53.7	31.9	54.8	
Tree utility to justify adoption of agroforestry practices in cocoa farms (frequency in % of farmers)	Food	60.3	75.4	80.0	69.1	–
	Fertilization	79.5	55.4	50.0	65.5	
	Shade tree	32.1	43.9	36.7	37.0	
	Medicinal tree	26.9	8.8	6.7	17.0	
	Lumber	15.4	10.7	26.7	15.8	
	Delimitation	2.6	17.5	16.7	10.3	
	Firewood	0.0	10.5	23.3	7.9	
	Other	1.3	0.0	0.0	0.6	
Reasons for rejecting agroforestry (% of farmers)	No space	0.0	26.0	45.2	29.7	*p* < 0.05 α
	Sufficient or excessive number of trees	23.1	18.0	21.0	20.3	
	Not important	53.9	18.0	3.2	18.1	
	Stunting growth or crop death	11.5	18.0	9.7	13.0	
	High humidity/shading	0.0	4.0	14.5	8.0	
	Lack of tree knowledge	11.5	10.0	0.0	5.8	
	No reason	0.0	2.0	1.6	1.5	
	Other	0.0	4.0	4.8	3.6	

α, Pearson chi-squared test.

### Determinants of cocoa agroforestry adoption

The results of the logistic regression model are presented in Table 5. The model summary shows that the model has a significant pseudo R2 value (Nagelkerke) of 0.114 and the likelihood ratio test is also significant (p < 0.001), suggesting that the model as a whole is statistically significant.

**Table 5:** Socioeconomic determinants affecting tree planting and cocoa agroforestry adoption in the “Espace Taï”.

Independent variables	Model summary			Average marginal effect		
	Estimate	Std. error	p-value	Estimate	Std. error	p-value
Constant	0.496	0.533	0.188			
Gender (1—Male)	2.553*	0.395	0.018	0.218*	0.089	0.014
Household head (1— Yes)	1.586	0.322	0.152	0.108	0.076	0.158
Cash crops	0.726*	0.132	0.015	− 0.073*	0.029	0.012
Mirid attacks (1—Yes)	2.141	0.480	0.112	0.172	0.103	0.097
Black pod attacks (1—Yes)	0.426	0.460	0.063	− 0.182*	0.088	0.039
Duration of residency	1.020*	0.010	0.037	0.005	0.002	0.032
Pseudo R^2^ (Nagelkerke)	0.114					
Prob > Chi^2^	0.000					
No. of observations	323					

*p < 0.05.

The AME estimates for the independent variables indicate that gender, number of cash crops, and duration of residency are significant predictors of cocoa agroforestry adoption. Specifically, being male (gender = 1) is positively associated with cocoa agroforestry adoption, with an estimated AME of 0.218 (p < 0.05). For every year of increase in the duration of residency, the probability of cocoa agroforestry adoption increases by 0.5%, with an estimated AME of 0.005 (p < 0.05). On the other hand, an increase in the number of cash crops is negatively associated with cocoa agroforestry adoption, with an estimated AME of − 0.073 (p < 0.05).

In addition, the table shows that the number of households experiencing black pod attacks has a negative effect on cocoa agroforestry adoption, with an estimated AME of − 0.182 (p < 0.05), while an increase in the number of male members of the household has a positive effect on cocoa agroforestry adoption, with an estimated AME of 2.553 (p < 0.05).

Overall, these results suggest that gender, number of cash crops, duration of residency, and incidence of black pod attacks are important factors influencing cocoa agroforestry adoption in the Espace Taï region.

## Discussion

### Socio-economic characteristics and cocoa agroforestry adoption

The findings of the current work revealed that 93% of farmers in the “*Espace Taï”* are migrants from other parts of the country as well as from other countries. This large proportion of non-local population is explained by the massive migration of new farmers attracted by the cocoa boom and prior cocoa farmers who moved from the eastern part of the country to the centre-west and then to the south-west parts searching for fertile endogenous soils in the primary forests. This massive movement which took place initially in the 1980s was termed by Ruf and Varlet^15^ as the cocoa pioneer front displacement. A second vague of cocoa farmers' migration occurred during the socio-political crisis between 2002 and 201^15,44^. The development of cocoa farmers' settlements in the vicinity of the TNP results in many instances in the encroachment on GFs and protected areas, such as in the Mount Péko National Park and the Goin-Debe forest reserve^4,45^.

The likelihood of adopting cocoa agroforestry may be influenced by the origin and ethnicity of the farmers, possibly because some of the migrants had prior experience with cocoa farming, including the benefits of keeping other tree species (shade, food, fuel and medicinal uses) within their cocoa orchards^46,47^.

A typical farmer is an adult, married, and illiterate and this has already been observed by Assiri et al.^48^. It has been shown that the education level positively improved the propensity to replant and adopt agroforestry^49^.

### Current cocoa farms characteristics and cropping practices

The average cocoa farms size was of 5.2 ha, which is lower than the 6.3 ha observed by Assiri et al.^48^ in the same area. The shrinkage of the cocoa farm size over time could be explained by the reduction of forest areas, and the farm inheritance between several heirs. Also, because of the different challenges encountered in cocoa farming and for diversification purposes, some farmers convert part of their cocoa orchards into alternative crops such as rubber and oil palm^50^.

Most farmers used unimproved planting material and other ineffective cropping practices leading to low average yields as already documented by Assiri et al.^48^ and Balineau et al.^51^, which is also due to climate change, and pest and disease pressure in the region. Good agricultural practices such as pruning, suckering, cutting, and sanitary harvest are critical to boosting cocoa productivity. Our findings showed that these practices are not timely and effectively implemented by most farmers, although doing so could reduce pest and disease impacts and increase productivity. The cocoa yield of 376.3 kg ha^−1^ found in the landscape is lower than the productivity of 2100–2400 kg ha^−1^ identified by research stations with hybrid plant materials^52^. The estimate of yield found in the study is close to the average of 400 kg ha^−1^ found in the same region^48,50^ and in traditional agroforestry systems of Colombia^53^. Low yields may be explained by the high use of non-selected plant materials, non-compliance with technical outreach, and the lack of farm maintenance as well as climate change, pest and disease impacts in the region. Additionally, cocoa trees generally become less productive as they age, and the age distribution of the trees in the study could potentially contribute to the observed lower yield. Interestingly, Adji et al.^54^ found that cocoa farms averaged 24.5 years in the region. This suggests that a significant proportion of the cocoa trees may be in their later stages of productivity, which could contribute to the overall decline in yield. In terms of harvesting patterns, cocoa pods are harvested monthly with the highest levels from September to December. Similar findings were observed in Ghana with high production occurring from October to February^55^.

Maintenance practices including pruning, suckering, cutting, and sanitary harvest are critical to boosting cocoa productivity. Our findings showed that farmers do not carry out timely and proper maintenance practices, which negatively affects cocoa beans’ quality. These practices are vital for minimizing the competition posed by pests and diseases and maximizing productivity. Pruning, for instance, has a positive effect on yields^23,56^ while also lowering the need for chemical fertilizers and pesticides. The low adoption of phytosanitary harvests (harvesting of infested and mummified pods, mistletoe, and water shoot) could reduce the productivity and the yield of cocoa plantations. Ndoumbe-Nkeng et al.^57^ highlighted that pod removal and phytosanitary harvests practices could enhance by 50% cherelles’ production and the number of ripe-healthy pods. Sanitation pruning reduces the contagion of black pod disease caused by *Phytophthora* spp.^57^. GAP recommend using pesticides and fertilizers to improve productivity and sustain yield^52,58–60^. The non-adoption of fertilizers can be explained by the high price of chemical fertilizers. In contrast, some low-income farmers use chicken droppings and compost which are costless as alternative options to boost the growth and the development of cocoa farms^61^. However, one of the challenges cocoa farmers faced is the financial cost of these maintenance practices which remain time-consuming. The low yield cannot allow farmers to drive more investments in soil inputs and other maintenance practices. With the scarcity of casual workers and funding coupled with the relatively huge mean size (5.2 ha) of cocoa farms, it would be challenging for farmers to perform solely these maintenance activities.

Although farmers are used to weeding manually their plantations, half adopted the use of herbicides in order to be overwhelmed by the abundance of weeds, the emergence of new and more aggressive species, and get accounted to labor unavailability^61^. Herbicide usage by smallholder farmers is led by the lack of workers and the low yield which cannot afford to pay anyone to help them. Farmers use herbicides and other practices like agroforestry to reduce labor costs that affect their revenues. Konlan et al.^62^ highlighted that glyphosate application significantly reduced weed management costs and increased the yield of three-year-old cocoa compared to manual weeding. Additionally, Armengot et al.^63^ revealed that cacao agroforestry systems offer higher labor returns compared to full-sun monocultures in Bolivia. However, regular usage of herbicides and pesticides may reduce the quality of cocoa beans by increasing pesticide residues and other harmful substances in beans^64^.

In cocoa orchards, mirid and black pod attacks targeting trees and pods are common and cause significant loss of productivity estimated at around 30–40% and 35%, respectively^65,66^. In the same way, the prevalence of CSSVD can lead to complete orchards’ destruction^65,66^. These pest and disease attacks could explain the low yield identified in the region.

Furthermore, the findings of this study indicate that a significant majority of cocoa orchards are cultivated under the shade of trees. This observation aligns with previous research that emphasized the widespread practice of growing cocoa under light to mid-level shade in Côte d'Ivoire^11,25^. In line with this, another study has underscored the influence of diverse factors, including social networks, ethnic groups, and geographic zones, on the presence and density of shaded trees within cocoa plantations^67^. Importantly, the implementation of agroforestry practices has demonstrated its potential to enhance both the yield and economic performance of cocoa agroforestry systems when compared to cocoa monoculture in Latin America^68,69^ and South Asia^70^.

### Drivers of cocoa agroforestry adoption by farmers

Our study revealed that the adoption of agroforestry is associated with key factors, including gender, length of residency, and the number of cash crops grown by the farmers. In a previous study, Owusu and Frimpong^71^ found that cocoa agroforestry adoption depends on age, gender, and household size and increased cocoa yield and thus household incomes. The gender differentiation could be explained by the fact the large majority of cocoa farmers are male who have more income to reinvest in the additional workload for farm diversification i.e. agroforestry practices. Indeed, the limited land rights access for women can lead to smaller land sizes and thus lower income generated compared to male farmers. Similar gender differentiation has been observed by different authors in Nigeria^72^, Ghana^71^, Cameroon^55^ and Indonesia^73^.

Although farmers value cocoa agroforestry in the region, most orchards are intercropped with tree species other than cocoa at an average low density (< 20 trees per ha) confirming the full sun monocropping system common in the study areas^74^. This low density is dominated by edible fruit species corroborating prior results that fruit and other food tree species were the dominant tree species in cocoa landscapes^11,74,75^. These low diverse agroforestry systems are comparable to the *Cabruca* systems in Brazil^76^ and also include species that can contribute to climate adaptation and mitigation, soil fertility improvement, and reduction of disease pressure^77,78^. These species can help cocoa farmers in diversifying their revenues and ensuring food security^79^, and to boost cocoa yield^75^. The difference in cocoa yields due to cocoa agroforestry adoption might be attributed to farmers age, soil fertility enhancement and soil moisture in cocoa agroforestry plantations. Despite the importance of cocoa agroforestry, more than 50% of the farmers indicated that they removed trees due from their farms to avoid competition for resources with cocoa trees and the spread of black pod disease and CSSVD following advice from extension services^74^. As an example, species such as *Psidium guajava* and *Cola nitida* are commonly removed by farmers due to nutrient competition for the former, and host of CSSVD for the latter species^74,80^, although no well-documented proof exists to confirm these reasons. The decision to remove trees may also be influenced by external factors, such as unauthorized logging activities that exert pressures and cause damages, which are not always effectively regulated by the forestry administration^11^.

More than 50% of the farmers indicated that they would agree to plant trees or adopt cocoa agroforestry using tree species such as legume, food, fruit, and shade trees. These findings are consistent with previous studies in West Africa as the diversity of shade tree diversity increases soil fertility and reduces the damage caused by cocoa plantation pests^78,81^. These studies provide evidence of the benefits associated with shade tree diversity in cocoa agroforestry. Additionally, cocoa agroforestry systems can help mitigate CSSVD severity^19^. The adoption of cocoa agroforestry systems with a minimum of 18 trees per hectare from 3 to 5 species can benefit from the certification schemes resulting in premium payment to the farmers^82^. Similarly, the REDD+ and biodiversity programs in the region have also contributed to the adoption of cocoa agroforestry^83^.

Despite the above benefits, about 45% of the farmers indicated that they do not intend to keep and plant more trees in their cocoa orchards due to tree tenure and the survival rate of newly planted trees. Recent studies conducted in Côte d’Ivoire revealed that more than 50% of trees found in cocoa farms are remnants of forests and encouraged sustainability initiatives and farmers to bet on natural regeneration^84,85^. It is worth noting that most farmers are unaware that the new forest code has transferred tree ownership from the state to individual farmers, granting them greater control over tree management decisions. Under the previous forest code, in effect until 2014, trees within or outside forests belonged to the state, allowing loggers to obtain permits for resource extraction. In addition, the new code has also put a substantial emphasis on the essential role of cocoa agroforestry in the restoration of the degraded forest and cocoa landscapes. This observation highlights the need for more proactive communication regarding the provisions of this new forest code to improve the adoption of cocoa agroforestry in the region.

Similarly, gender, the presence and the preference of tree species on cocoa farmland are among the factors that influence agroforestry adoption. Studies conducted in Nigeria^72^ and Côte d’Ivoire^11^ have shown that female groups are less likely to participate in agroforestry compared to their male counterparts. However, female farmers exhibit an interest in cocoa agroforestry, particularly in selling food and fruit trees like *Irvingia gabonensis* and *Ricinodendron heudelotii*, which can be integrated into cocoa plantations. While cocoa remains predominantly male-dominated, the involvement of female farmers in agroforestry activities within cocoa plantations provides them with opportunities to engage in income-generating activities and diversify their agricultural production^73,86^. Also, the length of residency significantly affects agroforestry adoption. Farmers with a longer residency may own larger properties with better environmental features^87^ as a result of their participation in various training and sensitization programs provided by extension services on tree adoption, which may enhance their adoption of cocoa agroforestry. Additionally, longer years of residence may be correlated with enhanced land tenure security, thereby fostering the adoption of agroforestry practices. Similar findings^11^ underscored the significance of secure land tenure in facilitating agroforestry adoption.

This study has certain limitations that should be addressed since they may influence the generalizability of the findings. The limited number of independent variables included in the study is one possible restriction. The study may have neglected other relevant variables that might impact farmers’ adoption thoughts by focusing on a certain set of criteria. As a result, the few drivers used in this study may have limited the discovery of all relevant factors influencing cocoa agroforestry adoption. Another disadvantage is that the study relied on farmers’ self-reported data. This raises the chance of response bias or reporting mistakes, which might undermine the validity of the results. Furthermore, the study was limited to a narrow geographical location, which may restrict its generalizability.

## Conclusions

The study provides valuable insights into the adoption of cocoa agroforestry practices in southwestern Côte d’Ivoire. Our findings shed light on the characteristics of cocoa farms, field practices, and cocoa agroforestry practices prevalent in the area. Notably, the majority of farmers in the region (86.2%) embrace cocoa agroforestry, with companion trees playing a significant role in providing food and shade, thereby contributing to orchard rehabilitation and sustainability, diversification of income streams, generating additional revenues, and contributing to environmental protection. The widespread adoption of cocoa agroforestry is a positive step towards enhancing ecosystem resilience, conserving biodiversity, and promoting a more sustainable cocoa industry in the region.

Moreover, the study identifies key factors that influence cocoa agroforestry adoption, including gender, the length of residency, the incidence of black pods attacks and the number of cash crops. These findings underscore the importance of tailoring cocoa agroforestry programs and policies to consider both socioeconomic and agronomic factors, meeting the needs and preferences of local farmers. In particular, initiatives should focus on increasing awareness of the new forest and land codes and the importance of preserving trees in cocoa farms.

The results of this study provide valuable insights into the ongoing efforts of promoting agroforestry practices in cocoa farms and emphasize the need for continued efforts to promote sustainable agriculture practices in Côte d’Ivoire. Such practices are essential for protecting the environment and natural resources while ensuring the long-term viability of the cocoa-forest sector. By taking into account farmers’ perceptions and preferences, and providing clear incentives for the adoption of cocoa agroforestry practices, these efforts can help to achieve a more sustainable future for agriculture in the region.

### Supplementary Information

Supplementary Tables.

## Data Availability

The dataset generated and analyzed during the current study is available in the Mendeley repository: [https://data.mendeley.com/datasets/bt2wbhdg3g/2].
