# Supplementary material for: Drivers of cocoa agroforestry adoption by smallholder farmers around the Taï National Park in southwestern Côte d’Ivoire
Source: Sci Rep. 2023 Aug 31;13:14309. doi: 10.1038/s41598-023-41593-5 (PMC10471588; doi:10.1038/s41598-023-41593-5)
Supplement: Supplementary file 1 — Supplementary Tables. [file 41598_2023_41593_MOESM1_ESM.docx]

# SUPPLEMENTARY MATERIAL

Table S1. Description of variables and multicollinearity detection in multinomial logistic regression model.

Generalized variance Inflation Factor (GVIF) showing multicollinearity (adjusted generalized variance inflation factors (AGVIF) > 2.5) between the independent variables in logistic regression model with agroforestry adoption as dependent variable. This was developed using the car package ^1^ in the R platform 3.6.0 ^2^

| **Variables** | **Data type** | **Data description** | **GVIF** | **df** | **AGVIF** |
| --- | --- | --- | --- | --- | --- |
| Gender of the respondent | Categorical | 1=Male; 0=Female | 1.5 | 1.0 | 1.2 |
| Age of the respondent | Numerical | [0-100] | 4.7 | 1.0 | 2.2 |
| Position of the respondent in the household | Categorical | 1=Head of the household; 0=Other members | 1.9 | 1.0 | 1.4 |
| Duration of residency | Numerical | [0-100] | 1.4 | 1.0 | 1.2 |
| Number of cultivated cash crops | Numerical | [1-10] | 1.2 | 1.0 | 1.1 |
| Presence of mirids | Categorical | 1=Yes; 0=No | 1.8 | 1.0 | 1.4 |
| Presence of blackpod disease | Categorical | 1=Yes; 0=No | 1.8 | 1.0 | 1.3 |
| Presence of Cocoa Swollen Shoot Virus Disease | Categorical | 1=Yes; 0=No | 1.1 | 1.0 | 1.1 |
| Tree species adoption | Categorical | 1=Yes; 0=No | 1.2 | 1.0 | 1.1 |
| Advice for tree planting | Categorical | 1=Yes; 0=No | 1.1 | 1.0 | 1.0 |
| Tree species removal | Categorical | 1=Yes; 0=No | 1.0 | 1.0 | 1.0 |

AGVIF = GVIF^(1/(2*df))

**Table S1 References**

1. Fox, J. & Weisberg, S. *An R Companion to Applied Regression*. (Sage, 2019).

2. R Core Team. *R: A language and environment for statistical computing*. (R Foundation for Statistical Computing, 2019).

Table S2. Planting material origin, seeding, density, and harvesting practices in cocoa plantations in the “Espace Taï”

| **Farm attribute** | | **East** | **North-west** | **South-west** | ***Espace Taï*** | **p-value** |
| --- | --- | --- | --- | --- | --- | --- |
| Origin of planting material (frequency in % of farmers) | Unselected plants | 95.3 | 99.1 | 90.1 | 95.1 | *-* |
|  | ANADER / CNRA | 14.2 | 5.7 | 20.9 | 13.2 |  |
| Seeding type (% of farmers) | Direct seeding | 73.6 | 69.4 | 78.0 | 73.4 | *n.s.* **α** |
|  | Polybag seedlings | 22.6 | 27.8 | 19.8 | 23.6 |  |
|  | On-farm direct seedlings | 3.8 | 2.8 | 2.2 | 3.0 |  |
| Planting density (% of farmers) | 1,333 trees ha^–1^ | 12.1 | 3.7 | 13.2 | 9.6 | *p* < 0.05 **α** |
|  | 2,000 trees ha^–1^ | 0.0 | 0.93 | 1.1 | 0.6 |  |
|  | Irregular density | 73.4 | 64.8 | 78.0 | 71.8 |  |
|  | Do not know | 14.5 | 30.6 | 7.7 | 18.0 |  |
| Harvest frequency (% of farmers) | Every fortnight | 12.9 | 9.3 | 16.5 | 12.7 | *p* < 0.05 **α** |
|  | Every 3 weeks | 16.1 | 7.4 | 9.9 | 11.5 |  |
|  | Each month | 63.7 | 49.1 | 36.3 | 51.1 |  |
|  | According to ripeness intensity | 7.3 | 34.3 | 37.4 | 24.7 |  |
| Annual harvest (no.) | Number of harvests | 9.5^a^ | 5.2^b^ | 8.5^c^ | 7.8 | *p* < 0.05 ***β*** |

n.s.: non-significant; **α:** Pearson chi-squared test; ***β***: Kruskal-Wallis chi-squared test

Means within a column with the same letter are not significantly different from each other at the 5% level

Table S3. Declaration of highest cocoa production months in the Espace Taï

| Month | Frequency (%) | | | |
| --- | --- | --- | --- | --- |
|  | East | North-west | South-west | Espace Taï |
| January | 2.4 | 5.6 | 5.5 | 4.3 |
| February | 2.4 | 5.6 | 6.6 | 4.6 |
| March | 0.8 | 4.6 | 7.7 | 4.0 |
| April | 0.8 | 4.6 | 9.9 | 4.6 |
| May | 1.6 | 4.6 | 12.1 | 5.6 |
| June | 3.2 | 4.6 | 22.0 | 9.0 |
| July | 3.2 | 7.4 | 30.8 | 12.4 |
| August | 10.5 | 26.9 | 44.0 | 25.4 |
| September | 39.5 | 40.7 | 50.5 | 43.0 |
| October | 79.0 | 72.2 | 74.7 | 75.5 |
| November | 77.4 | 60.2 | 38.5 | 60.7 |
| December | 54.8 | 71.3 | 48.4 | 58.5 |

Table S4. Cocoa maintenance practices in the Espace Taï

| Cocoa maintenance | Frequency (%) | | | |
| --- | --- | --- | --- | --- |
|  | East | North-west | South-west | Espace Taï |
| Cutting suckers and offshoots | 95.2 | 93.5 | 71.4 | 87.9 |
| Pruning | 79.8 | 83.3 | 86.8 | 83.0 |
| Coppicing | 60.5 | 50.0 | 69.2 | 59.4 |
| Sanitary harvesting | 4.8 | 0.9 | 0.0 | 2.2 |
| Staking | 0.0 | 1.9 | 0.0 | 0.6 |

Table S5. Weeding practices in cocoa plantations in the Espace Taï

| **Weeding practices** | | **East** | **North-west** | **South-west** | **Espace Taï** | **p-value** |
| --- | --- | --- | --- | --- | --- | --- |
| Type of weeding (frequency in % of farmers) | Manual | 100 | 94.4 | 100 | 98.0 | - |
|  | Chemical | 59.4 | 62.0 | 46.7 | 56.6 |  |
| Number of weeding (no.) | Manual | 2.5 | 2.4 | 2.5 | 2.4 | *n.s.* ***β*** |
|  | Chemical | 1.3^a^ | 1.8^b^ | 1.5^a^ | 1.5 | *p* < 0.05 ***β*** |

n.s.: non-significant; **α:** Pearson chi-squared test; ***β***: Kruskal-Wallis chi-squared test

Means within a column with the same letter are not significantly different from each other at the 5% level

Table S6. Fertilization practices in cocoa plantations in the Espace Taï

| Fertilization practices | | East | North-west | South-west | Espace Taï | *p*-value |
| --- | --- | --- | --- | --- | --- | --- |
| Fertilizer application (% of farmers) | No | 25.8 | 32.4 | 26.4 | 28.2 | *n.s.* **α** |
|  | Yes | 74.2 | 67.6 | 73.6 | 71.8 |  |
| Type of fertilizer (frequency in % of farmers) | Chemical fertilizer | 91.5 | 90.4 | 95.7 | 92.4 | - |
|  | Compost / chicken droppings | 12.2 | 13.7 | 10.0 | 12.0 |  |
|  | Organic fertilizer | 9.8 | 11.0 | 12.9 | 11.1 |  |
|  | Foliar fertilizer | 13.4 | 0.0 | 4.3 | 6.2 |  |
| Quantity of fertilizer (t) | Chemical fertilizer | 0.9 | 1.0 | 0.9 | 0.9 | *n.s.* ***β*** |
|  | Organic fertilizer | 5.7 | 9.0 | 4.3 | 6.4 | *n.s.* ***β*** |
|  | Foliar fertilizer | 0.2 | - | 0.2 | 0.2 | *n.s.* ***β*** |
|  | Compost / chicken droppings | 0.5^a^ | 1.4^ab^ | 1.0^b^ | 1.0 | *p* < 0.05 ***β*** |
| Fertilized area (ha) | Chemical fertilizer | 3.2^a^ | 3.5^ab^ | 4.2^b^ | 3.7 | *p* < 0.05 ***β*** |
|  | Organic fertilizer | 3.4 | 3.9 | 4.3 | 3.9 | *n.s.* ***β*** |
|  | Foliar fertilizer | 3.6 | - | 6.0 | 4.1 | *n.s.* ***β*** |
|  | Compost / chicken droppings | 2.2 | 2.4 | 4.3 | 2.8 | *n.s.* ***β*** |

n.s.: non-significant; **α:** Pearson chi-squared test; ***β***: Kruskal-Wallis chi-squared test

Means within a column with the same letter are not significantly different from each other at the 5% level

Table S7. Pesticide usage practices in cocoa plantations in the Espace Taï

| Pesticide uses | | East | North-west | South-west | Espace Taï | *p*-value |
| --- | --- | --- | --- | --- | --- | --- |
| Pesticide application (% of farmers) | No | 5.6 | 7.4 | 15.4 | 9.0 | *p* < 0.05 **α** |
|  | Yes | 94.4 | 92.6 | 84.6 | 91.0 |  |
| Pesticide usage | Area (ha) | 4.7^a^ | 4.1^b^ | 5.3^a^ | 4.6 | *p* < 0.05 ***β*** |

n.s.: non-significant; **α:** Pearson chi-squared test; ***β***: Kruskal-Wallis chi-squared test

Means within a column with the same letter are not significantly different from each other at the 5% level

Table S8. Sanitary status of cocoa plantations in the Espace Taï

| **Sanitary status** | | East | North-west | South-west | Espace Taï | *p*-value |
| --- | --- | --- | --- | --- | --- | --- |
| Mirid attack (% of farmers) | No | 11.3 | 15.7 | 6.6 | 11.5 | *n.s.* **α** |
|  | Yes | 88.7 | 84.3 | 93.4 | 88.5 |  |
| Impact of mirid attacks (% of farmers) | Low | 34.3 | 17.6 | 45.9 | 32.4 | *p* < 0.05 **α** |
|  | Medium | 31.3 | 37.4 | 30.6 | 33.1 |  |
|  | High | 34.3 | 45.1 | 23.5 | 34.5 |  |
| Black pod attack (% of farmers) | No | 19.4 | 13.0 | 7.7 | 13.9 | *p* < 0.05 **α** |
|  | Yes | 80.6 | 87.0 | 92.3 | 86.1 |  |
| Impact of black pod attacks (% of farmers) | Low | 36.0 | 22.3 | 41.7 | 33.0 | *p* < 0.05 **α** |
|  | Medium | 34.8 | 40.4 | 39.3 | 38.2 |  |
|  | High | 29.2 | 37.2 | 19.0 | 28.8 |  |
| CSSVD suspicion (% of farmers) | No | 80.6 | 44.4 | 89.0 | 70.9 | *p* < 0.05 **α** |
|  | Yes | 19.4 | 55.6 | 11.0 | 29.1 |  |
| CSSVD signs (frequency in % of farmers) | Rapid and generalized death of cocoa trees | 91.7 | 76.7 | 70.0 | 79.8 | - |
|  | Red ban vein on young leaves | 16.7 | 63.3 | 70.0 | 52.1 |  |
|  | Small rounded pods | 20.8 | 50.0 | 40.0 | 41.5 |  |
|  | Swelling of roots | 8.3 | 45.0 | 30.0 | 34.0 |  |
|  | Swelling of tips, shoots and twigs | 0.0 | 41.7 | 50.0 | 31.9 |  |
|  | Mosaic on aged leaves | 4.2 | 33.3 | 40.0 | 26.6 |  |

n.s.: non-significant; **α:** Pearson chi-squared test.

Table S9. Agroforestry adoption advice in cocoa plantations of the Espace Taï

| **Agroforestry advice** | | East | North-west | South-west | Espace Taï | *p*-value |
| --- | --- | --- | --- | --- | --- | --- |
| Advice for tree planting (% of farmers) | No | 72.6 | 58.3 | 57.1 | 65.5 | *p* < 0.05 **α** |
|  | Yes | 27.4 | 41.7 | 42.9 | 36.5 |  |
| Extension services advised for tree planting (frequency in % of farmers) | ANADER | 83.3 | 65.2 | 91.3 | 79.7 | - |
|  | Friends | 11.1 | 26.1 | 34.8 | 25.0 |  |
|  | Parents | 55.6 | 13.0 | 8.7 | 23.4 |  |
|  | CNRA | 0.0 | 4.3 | 56.5 | 21.9 |  |
|  | Certification schemes | 11.1 | 0.0 | 0.0 | 3.1 |  |
|  | Other | 0.0 | 13.0 | 8.7 | 7.8 |  |

CNRA: Centre National de Recherche Agronomique, ANADER: Agence National de Développement Rural

n.s.: non-significant; **α:** Pearson chi-squared test.

Table S10. On-farm tree management in cocoa plantations of the Espace Taï

| Tree species management | | East | North-west | South-west | Espace Taï | *p*-value |
| --- | --- | --- | --- | --- | --- | --- |
| Tree removal (% of farmers) | No | 43.5 | 44.4 | 42.9 | 43.7 | *n.s.* **α** |
|  | Yes | 56.5 | 55.6 | 57.1 | 56.3 |  |
| Reasons for tree removal (frequency in % of farmers) | Too much shade | 21.9 | 31.7 | 36.5 | 29.5 | - |
|  | Incompatible / competing with cocoa trees | 31.2 | 35.0 | 17.3 | 28.4 |  |
|  | High number of trees | 12.5 | 21.7 | 34.6 | 22.2 |  |
|  | Shelters for pests | 10.9 | 11.7 | 3.8 | 9.1 |  |
|  | Damage of loggers | 9.4 | 0.0 | 0.0 | 3.4 |  |
|  | Source of black pod | 4.7 | 0.0 | 5.8 | 3.4 |  |
|  | Wood for construction | 6.2 | 0.0 | 0.0 | 2.3 |  |
|  | Too big | 4.7 | 1.7 | 0.0 | 2.3 |  |
|  | Other | 14.1 | 8.3 | 9.6 | 10.8 |  |

n.s.: non-significant; **α:** Pearson chi-squared test.
